# β-glucan-coupled superparamagnetic iron oxide nanoparticles induce trained immunity to protect mice against sepsis

**DOI:** 10.7150/thno.64874

**Published:** 2022-01-01

**Authors:** Yuchen Pan, Jingman Li, Xiaoyu Xia, Jiali Wang, Qi Jiang, Jingjing Yang, Huan Dou, Huaping Liang, Kuanyu Li, Yayi Hou

**Affiliations:** 1 State Key Laboratory of Pharmaceutical Biotechnology, Division of Immunology, Medical School, Nanjing University, Nanjing 210093, China.; 2 State Key Laboratory of Trauma, Burns and Combined Injury, Research Institute of Surgery, Daping Hospital, The Army Medical University, Chongqing 400042, China.; 3 Jiangsu Key Laboratory of Molecular Medicine, Nanjing 210093, China.

**Keywords:** Sepsis, Macrophages, Trained immunity, β-glucan, SPIO

## Abstract

**Background:** Innate immune memory, also termed “trained immunity”, is thought to protect against experimental models of infection, including sepsis. Trained immunity via reprogramming monocytes/macrophages has been reported to result in enhanced inflammatory status and antimicrobial activity against infection in sepsis. However, a safe and efficient way to induce trained immunity remains unclear.

**Methods:** β-glucan is a prototypical agonist for inducing trained immunity. Ferumoxytol, superparamagnetic iron oxide (SPIO) with low cytotoxicity, has been approved by FDA for clinical use. We synthesized novel nanoparticles BSNPs by coupling β-glucan with SPIO. BSNPs were further conjugated with fluorescein for quantitative analysis and trace detection of β-glucan on BSNPs. Inflammatory cytokine levels were measured by ELISA and qRT-PCR, and the phagocytosis of macrophages was detected by flow cytometry and confocal microscopy. The therapeutic effect of BSNPs was evaluated on the well-established sepsis mouse model induced by both clinical *Escherichia coli* (*E. coli*) and cecal ligation and puncture (CLP).

**Results:** BSNPs were synthesized successfully with a 3:20 mass ratio of β-glucan and SPIO on BSNPs, which were mainly internalized by macrophages and accumulated in the lungs and livers of mice. BSNPs effectively reprogrammed macrophages to enhance the production of trained immunity markers and phagocytosis toward bacteria. BSNP-induced trained immunity protected mice against sepsis caused by *E. coli* and CLP and also against secondary infection. We found that BSNP treatment elevated Akt, S6, and 4EBP phosphorylation, while mTOR inhibitors decreased the trained immunity markers and phagocytosis enhanced by BSNPs. Furthermore, the PCR Array analysis revealed *Igf1*, *Sesn1*, *Vegfa,* and *Rps6ka5* as possible key regulators of mTOR signaling during trained immunity. BSNP-induced trained immunity mainly regulated cellular signal transduction, protein modification, and cell cycle by modulating ATP binding and the kinase activity. Our results indicated that BSNPs induced trained immunity in an mTOR-dependent manner.

**Conclusion:** Our data highlight that the trained immunity of macrophages is an effective strategy against sepsis and suggest that BSNPs are a powerful tool for inducing trained immunity to prevent and treat sepsis and secondary infections.

## Introduction

Sepsis is a major socioeconomic burden worldwide 1-3 and defined as a clinical syndrome characterized by multiple organ failure due to dysregulated host response to infection 4-6. Despite rapid advances in early diagnosis and the use of antibiotics and personalized treatment, sepsis still has a mortality rate of up to 25% 7-8. Moreover, coronavirus disease 2019 (COVID-19) could be better described as “viral sepsis” 9-10, since some patients with COVID-19 met the diagnostic criteria for septic shock and sepsis 11-12. Therefore, we focused on developing preventive measures or broad-spectrum therapy for sepsis.

Innate immune response boosted after experiencing initial stimulus, termed as “trained immunity”, is a powerful defense against infection, regardless of bacteria, fungi or virus 13. β-glucan-mediated trained immunity in cells induced the epigenetic reprogramming of myeloid cells, especially monocytes and macrophages 14, resulting in an enhanced inflammatory status 15 and antimicrobial activity 16. Trained immunity also protected dogs against rabies 17 and mice against a lethal dose of *Candida albicans* 18 and *Mycobacterium tuberculosis* 19. As trained immunity can lead to nonspecific protection from re-infection, it may provide a new tool for preventing and treating sepsis 20. Thus, it is essential to explore secure and efficient ways to induce trained immunity.

The emergence of nanotechnology offers an opportunity to use nanoparticles as carriers for vaccine and drug delivery 21-23. Ferumoxytol, superparamagnetic iron oxide (SPIO) with low cytotoxicity, has been approved by FDA for clinical use as an iron supplement 24-25. After coupling with other materials, SPIO could be used for the diagnosis and treatment of lung 26, liver 27-28, and colon cancers 29. Disruption of iron homeostasis is a common feature of sepsis, characterized by decreased serum iron and transferrin levels 30-31. Lactoferrin supplementation reduced late-onset sepsis in preterm infants without adverse effects 32. Our previous studies showed that SPIO alleviated LPS-induced sepsis in mice by promoting macrophages to secrete IL-10 33. However, it was still unclear whether proper engineering of SPIO with β-glucan could efficiently induce the trained immunity of macrophages against infection in sepsis.

In the current study, we synthesized β-glucan-coupled SPIO nanoparticles (BSNPs) and investigated their protective effect in the clinical *E. coli*- and CLP-induced sepsis mouse model. We found that BSNP-induced trained immunity improved macrophage phagocytosis of bacteria and promoted macrophages to produce inflammatory factors to kill bacteria in an mTOR-dependent manner. Importantly, BSNP treatment could prevent sepsis and also protect mice against secondary infection. Our data indicated BSNPs as a potential strategy for sepsis prevention and treatment by promoting trained immunity.

## Materials and methods

### Reagents

LPS (E. *coli* O55:B5) and β-glucan (CAS: 9041-22-9) were purchased from Sigma (Shanghai, China) and prepared in double distilled water (ddH_2_O). M-CSF (Novoprotein, Shanghai, China) for BMDM was prepared in ddH_2_O. Rapamycin (HY-10219), Torkinib (HY-10474) and WAY600 (HY-15272) were purchased from MedChemExpress (MCE, Monmouth Junction, NJ, USA) and prepared in DMSO. The antibodies used for flow cytometry (supplemental Table S1) and PCR Assay plates (Wcgene Biotech, Shanghai, China) were all purchased from fcmacs (Nanjing, China). Superparamagnetic iron oxide nanoparticles (SPIO, Ferumoxytol) was kindly provided by professor Ning Gu from Southeast University.

### Cells and Culture Conditions

Raw264.7 cells and mice peritoneal macrophages were cultured in DMEM (Gibico, Grand iSland, NY, USA) containing 10% FBS (Gibico), 1% penicillin and streptomycin (100 μg/mL; Gibco BRL, USA), at 37 °C in a humidified atmosphere with 5% CO_2_.

### Bacterial Strain

The clinical strains of E. *coli* (E. *coli^a^* for short) were isolated from human clinical specimens, and identified by the Medical Laboratory Center of Zhongda Hospital in Nanjing, Jiangsu, China. Bacterial strains were stored at -80 °C and prepared in LB Medium or LB-Agar medium before use.

### Mice

Female ICR mice at 6-8 weeks old were obtained from Sino-British SIPPR/BK Lab. Animal Ltd (Shanghai, China). They were acclimatized for 5-7 days before molding. All procedures involving animals were in strict accordance with protocols that approved by the Research Ethics Committee of Nanjing University. Mice were housed in specific pathogen-free conditions at the Nanjing University Animal Care Commission. At the end of the experiment, mice were terminated humanely.

### Experimental sepsis

Early removal criteria: 1) Weight loss: loss of 20% of their baseline body weight; 2) Weakness/ inability to obtain feed or water: inability or extreme reluctance to stand/ambulate for 24 hours.

E. *coli^a^* grown to mid-exponential phase was harvested, washed and resuspended with normal saline. Mice were injected intraperitoneally (i.p.) with 0.2 mL bacterial suspension. Mortality was recorded after mice injected with lethal dose of E. *coli^a^*.

Polymicrobial sepsis was induced by cecal ligation and puncture (CLP). Briefly, after anesthesia with pentobarbital, mice were placed on electric homeothermic blanket to maintain body temperature at 36.5 °C. The cecum was ligated below the ileocecal valve through a small abdominal incision and subjected to a single “through-and-through” perforation (20-gauge needle). Then the abdominal incision was closed in layers. Mice in sham group underwent the same surgical without ligation and puncture on cecum. Body weight was recorded every day before sacrifice.

### Trained immunity *in vivo*

Mice were injected intraperitoneally with normal saline (group c), β-glucan (500 μg, group b), SPIO (150 μg, group s), β-glucan (500 μg) combined with SPIO (150 μg) (group bs) or BSNPs (SPIO 150 μg composited with β-glucan 22.5 μg, group n) twice a week before sacrifice or *E. coli^a^* injection. Mice from each group injected with *E. coli^a^* were marked as c-E, b-E, s-E, bs-E or n-E.

### Trained immunity *in vitro*

Mice macrophage cell line Raw264.7 cells were treated for 24 h with β-glucan (30 μg/mL, group b), SPIO (200 μg/mL, group s), β-glucan (30 μg/mL) combined with SPIO (200 μg/mL) (group bs) or BSNP (SPIO 200 μg composited with β-glucan 30 μg/mL, group n). Then cells were washed with PBS for 3 times, and cultured with fresh medium for 5 days before use.

### Histology and Tissue Injury Scoring

Lung and liver tissue sections were stained with hematoxylin and eosin (H&E) and observed under a light microscope. Based on the presence of exudates, hyperemia/congestion, infiltration of inflammatory cells, morphological changes of lung tissue were scored as nil (0), mild (+1), moderate (+2), or severe (+3) injury 34-35. Based on the vacuolization, sinusoidal congestion, and hepatocyte necrosis, liver injuries were scored from 0 to 4 36. The sum of scores of different animals was averaged.

### Synthesis of BSNPs

Superparamagnetic iron oxide nanoparticles (SPIO; 20 mg, containing carboxyl groups on the surface) were first dissolved in a solution of 20 mL ddH2O containing 1-ethyl-3-(3-dimethylaminopropyl) carbodiimide hydrochloride (EDC; 22 mg) (Bidepharm, Shanghai, China). The mixture was then stirred at room temperature for 30 min and β-glucan (4 mg) was then added to the mixture and stirred overnight at room temperature. Thereafter, the reaction mixture was concentrated and then added excess ethanol to obtain crude BSNPs. The crude products were resuspended with ddH_2_O, washed with excess ethanol, and then dried under reduced pressure.

### Synthesis of BSNP-RhB or BSNP-NIR

First, β-glucan (10 mg) and Rhodamine B isothiocyanate (RhB; 0.5 mg; CAS: 36877-69-7) or NIR-797 (NIR; 0.5 mg; CAS: 152111-91-6) were added into a 10 mL flask with 5 mL dimethyl sulfoxide (DMSO; Sigma). The mixture was stirred overnight at room temperature, and then diluted with 20 mL deionized water. The diluted mixture was dialyzed against 2 L of ddH_2_O in a 3.5 kDa MWCO for two days to remove the unreacted RhB or NIR. The products were dried under reduced pressure to obtain fluorescence-glucan and used to synthesize fluorescence-BSNPs (BSNP-RhB or BSNP-NIR) with the same protocol as BSNPs.

### Quantitative Analysis of β-glucan on BSNPs

BG-RhB was diluted with ethanol to perform five two-fold serial dilutions in separate tubes. After diluting, the BG-RhB concentrations are 0.4 mg/mL, 0.2 mg/mL, 0.1 mg/mL, 0.05 mg/mL and 0.025 mg/mL, respectively. Ethanol serves as the zero standard (0 mg/mL). Full-function Fluorescence Spectrometer (FLS920, Edinburgh Instruments, UK) was used to detect the spectrum, create fit curve and calculate integration area. Plot the standard curve with concentration on the x-axis and integration area on the y-axis to create standard formula (Supplemental Table S2). During synthesis BSNP-RhB, a certain volume of washing ethanol was collected. The washing ethanol was then diluted with ethanol to perform different samples to make it more accurate. Spectrum and integration area of samples were detected with fluorescence spectrometer. The standard formula was used to calculate the concentration of each sample.

The theoretical density of molecules on nanoparticle surface ***ρ*** can be calculated via:



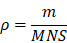



where the mass of β-glucan ***m*** can be calculated via standard formula, the average molecular weight of glucan*** M*** that we used was about 30,000, the number of nanoparticles ***N*** can be determined by Dynamic Light Scattering, and the superficial area of nanoparticles ***S*** can be calculated with average diameter that determined by Transmission Electron Microscopy (TEM). The theoretical density of glucan on BSNPs surface is 0.005 mol/m^2^, and molecules of glucan on BSNPs surface is around 3000 /nm^2^.

### Bio-distribution analysis of BSNPs

BSNP-NIR was intraperitoneally injected into mice. After 24 h, the *ex vivo* imaging of major organs was performed using *In vivo* Imaging System (LB 983 NC100, Berthold, Germany). BSNP-RhB was intraperitoneally injected into mice, and peritoneal macrophage was washed with PBS for two times to obtain single cell suspension for fluorescent antibody staining. BSNPs uptake by macrophages were measured by confocal microscope or flow cytometry.

### Enzyme Linked Immunosorbent Assay (ELISA)

TNFα, IL-1β and IL-6 were measured using ELISA kit according to the manufacturer protocol (TNFα and IL-6: BioLegend; IL-1β: R&D). Cells were lysed with ddH_2_O. Supernatants and lysates were collected and stored at -80 °C until assayed.

### Western Blot

Proteins were exacted with RIPA lysis buffer (Beyotime, Shanghai, China) as protocol described. After electrophoresis on SDS-PAGE, proteins were transferred onto PVDF membranes (Millipore). The antibodies used for western blot and immunofluorescence were all purchased from Cell Signaling Technologies (CST, Beverly, MA, USA; Supplemental Table S3).

### Immunofluorescence

For immunofluorescence analysis, cells were seed on the plates pre-coated with cover slides. At the end of experiment, 4% paraformaldehyde (PFA) was added onto each pore for 10 min, and then wash cells with ddH_2_O at least 3 times. The cover slides were then placed on the glass slide with anti-fluorescence quenching agent. Fluorescence images were obtained using confocal microscope (FV3000, Olympus, Japan/Stellaris, Leica, Germany).

### RNA Extraction and Quantitative Real-Time PCR

Total RNA was isolated using Trizol Reagent (Vazyme, Nanjing, China) according to the manufacturer's instructions. Collected mRNA was reverse-transcribed to cDNA using HiScript ® Ⅱ Q RT SuperMix kit (Vazyme). Real-time PCR assay was then performed using SYBR green dye (Invitrogen) on StepOne sequence detection system (Applied Biosystems, Waltham, MA, USA). Relative abundance of genes was calculated by using 2^-ΔΔCT^ formula, with β-actin as an internal control. The sequences of the qRT-PCR primers are provided in Supplemental Table S4. PCR Array assay was performed according to the manufacturer's protocol (Wcgene Biotech, Shanghai, China) on StepOne sequence detection system.

### Statistical analysis

All of the values presented on the graphs are given as means ± S.E.M. ANOVA and unpaired Student's t-tests were used to analyze the statistical significance. Statistical differences were defined as significant for *p < 0.05 and highly significant for **p < 0.01 and ***P < 0.001. GraphPad Prism5 Demo (GraphPad Software Inc., La Jolla, CA, USA) was used for statistical analysis.

## Results

### Combination of β-glucan and SPIO induces trained immunity to protect mice against sepsis

**Figure 1:**
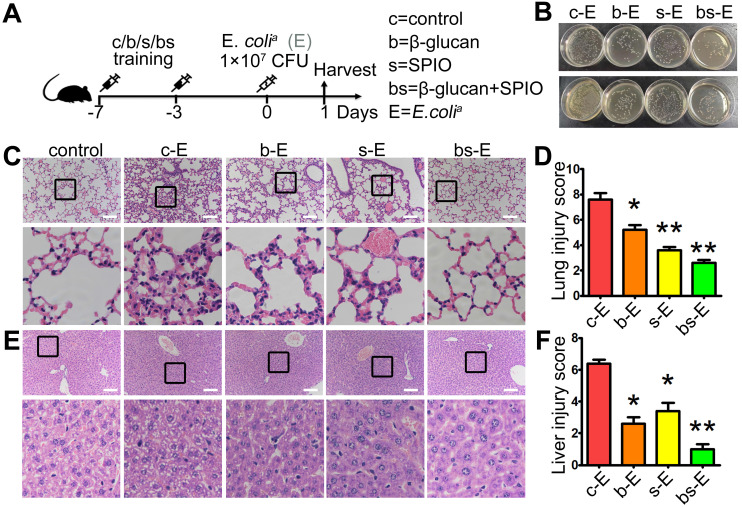
**Trained immunity prevents mice against sepsis *in vivo*. (A)** Schematic of sepsis after trained immunity experimental setup (n = 5). **(B)** Blood (upper) and peritoneal lavage fluid (below) from mice 24 h after infection was plated for 16 h. Representative plate shows bacterial colonies. Representative H&E staining of **(C)** lung sections or **(E)** liver sections. Scale bars, 100 µm. Histological injury of **(D)** lungs and **(F)** livers in different groups scored as described in Materials and Methods. Data are shown as mean ± SEM (n = 5). *P < 0.05; **P < 0.01.

We determined the therapeutic effect of SPIO and β-glucan to induce trained immunity against sepsis by using the well-established sepsis mouse model. Mice injected with normal saline (group c-E), β-glucan (group b-E), SPIO (group s-E), or β-glucan combined with SPIO (group bs-E) twice a week were then administered with a non-lethal dose of clinical *Escherichia coli* (*E. coli^a^*) (Figure 1A); mice injected intraperitoneally with normal saline were used as controls. Cloning assay showed that bacterial burden of peripheral blood and peritoneum was slightly decreased in mice treated with SPIO, while significantly decreased in mice treated with β-glucan or with the combination of β-glucan and SPIO (Figure 1B). Moreover, treatment with combined β-glucan and SPIO decreased the inflammatory cell infiltration and hyperemia in the alveolar walls (Figure 1C, D). The treatment also decreased the vacuolization and sinusoidal congestion in the liver (Figure 1E, F). These data revealed that the treatment with combined β-glucan and SPIO was superior to β-glucan or SPIO alone in preventing mice against sepsis.

### Synthesis and characterization of BSNPs

**Figure 2:**
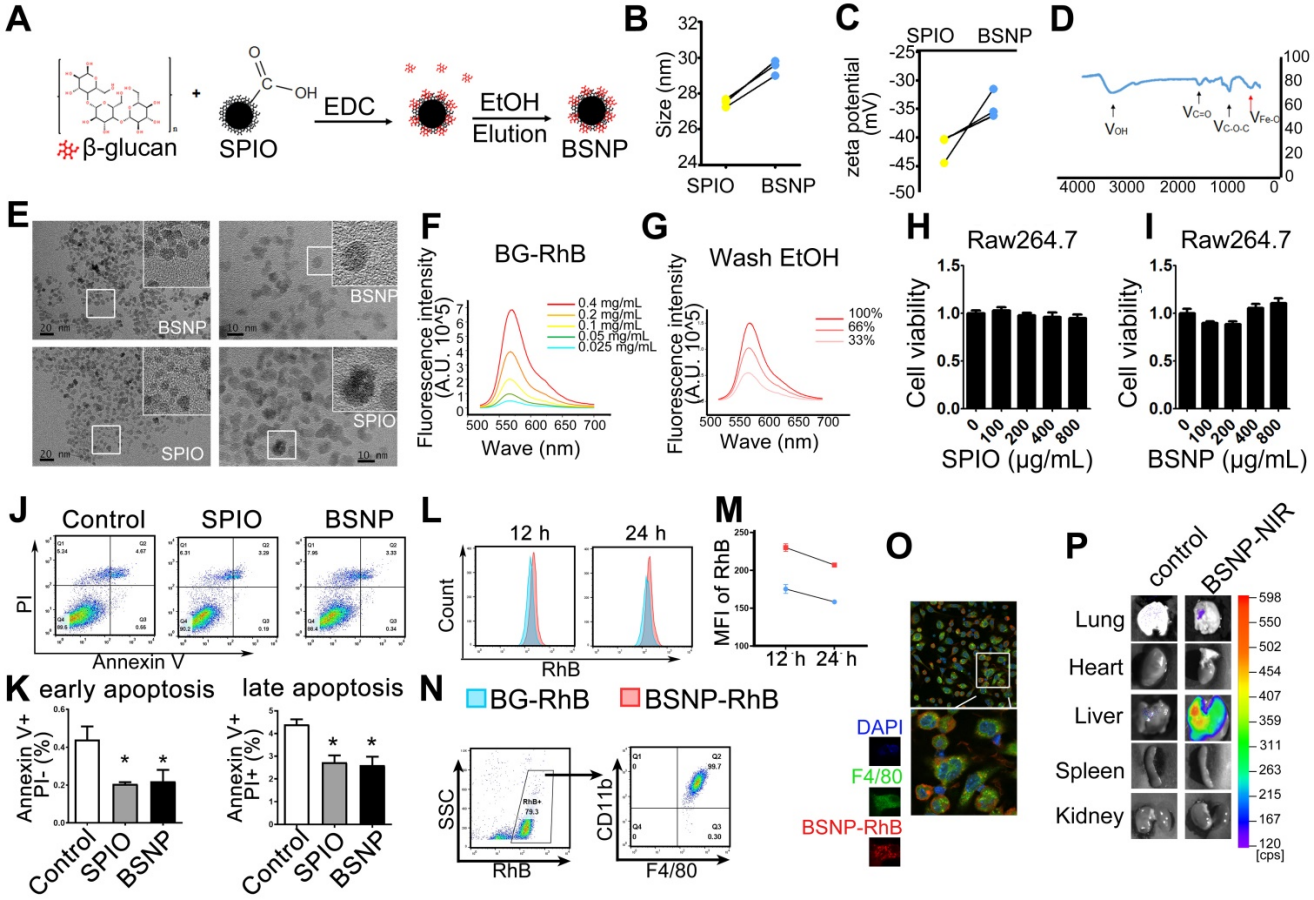
** Characteristic and cytotoxicity of BSNPs. (A)** Schematic of BSNPs synthesis. **(B)** Hydrate particle size and **(C)** zeta potential of SPIO and BSNPs. **(D)** Fourier transform infrared spectroscopy profile of BSNPs. **(E)** TEM images of SPIO and BSNPs. Scale bar, (left) 20 nm, (right) 10 nm. **(F)** Spectrum of fluorescence-glucan (BG-RhB) and **(G)** washing ethanol of fluorescence-BSNPs (BSNP-RhB) with different concentration detected as described in Materials and Methods. **(H-I)** Raw264.7 cells were treated with different concentration of SPIO or BSNPs for 24 h, cell viability was measured by CCK-8 assay. **(J-K)** Raw264.7 cells were treated with SPIO at 200 μg/mL for 24 h and cell apoptosis was measured by flow cytometry. **(L-N)** Mice were injected intraperitoneally with BG-RhB or BSNP-RhB and then sacrificed for peritoneal cells. The fluorescence intensity was measured by flow cytometry at 12 h and 24 h and **(O)** confocal microscope at 24 h. **(P)** Mice were injected intraperitoneally with BSNP-NIR for 24 h. The *ex vivo* imaging of major organs was carried out on the *In vivo* Imaging System. Data are shown as mean ± SEM (n = 3). *P < 0.05.

Next, we synthesized β-glucan coupled with SPIO (BSNPs) to optimize treatment, test application of nanomaterials, and provide a convenient and visual basis for the systemic delivery of β-glucan (Figure 2A). Dynamic Light Scattering showed that the hydration particle size of BSNPs was slightly increased to 30 nm (Figure 2B), and its zeta potential was -34 mV (Figure 2C, Supplemental Table S5). Fourier transform infrared spectroscopy displayed the characteristic peaks of β-glucan and iron oxide core in BSNPs (Figure 2D). TEM images revealed a homogeneous dispersion of spherical BSNPs with an average diameter of 8 nm, approximately the same as SPIO (Figure 2E). These data confirmed the successful synthesis of BSNPs.

We prepared fluorescence-labeled β-glucan (BG-RhB) to synthesize fluorescence-labeled BSNPs (BSNP-RhB) for the quantitative analysis of β-glucan. The spectrum of different concentrations of BG-RhB (Figure 2F) and the eluted ethanol containing the unbound BG-RhB were determined by Full-function Fluorescence Spectrometry (Figure 2G) (Supplemental Table S2). The mass ratio of β-glucan and SPIO on BSNP was 3:20, as calculated by the standard curve. When Raw264.7 cells were treated with different concentrations of SPIO or BSNPs for 24 h, cell viability was not affected even under the highest concentration at 800 μg/mL (Figure 2H-I). Flow cytometry showed that apoptosis of Raw264.7 cells was slightly decreased after SPIO and BSNP treatment (Figure 2J-K).

Mice were intraperitoneally injected with BG-RhB or BSNP-RhB to monitor the efficiency of macrophage internalization. The mice were then sacrificed to obtain peritoneal macrophages, and fluorescence intensity was determined by flow cytometry. High fluorescence intensity was detected in the BSNP-RhB group at 12 h and 24 h after BSNPs treatment, slightly declining in both groups at 24 h (Figure 2L-M). Almost all BSNP-RhB positive cells were CD11b+ F4/80+ macrophages (Figure 2N-O), indicating that the macrophages could ingest more BSNPs that were degraded slower than β-glucan. Moreover, *ex vivo* imaging of dissected tissues showed localization of BSNPs in the lungs and livers of septic mice (Figure 2P). The images from confocal microscopy revealed colocalization of F4/80+ macrophages and BSNP-RhB in the liver (Supplementary Figure S1). These data verified that BSNPs could accumulate in the damaged organs and be ingested by macrophages efficiently.

### BSNP-induced trained immunity promotes immune responses of macrophages

**Figure 3:**
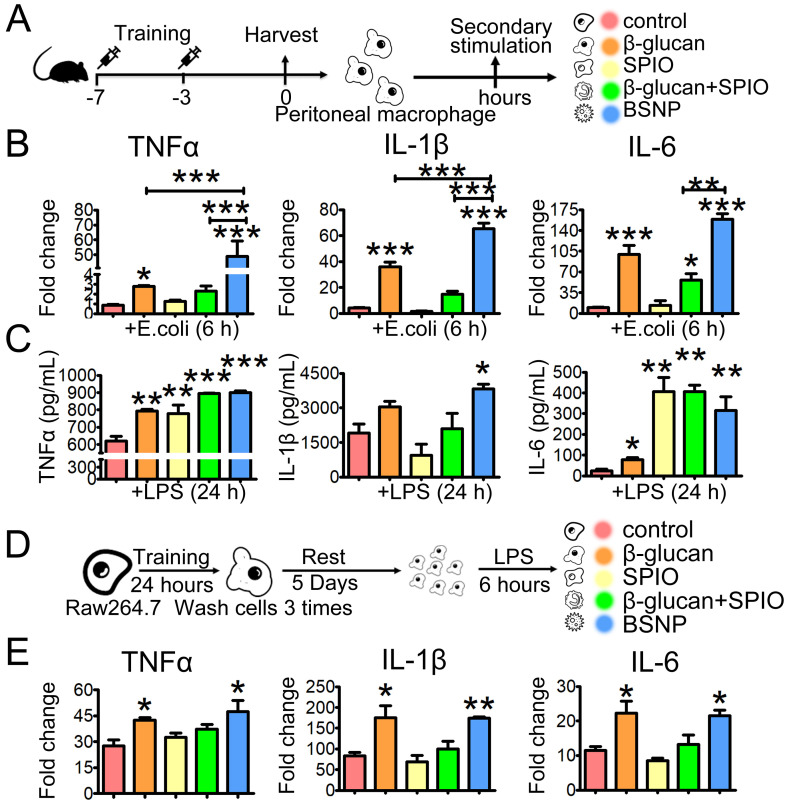
** Trained immunity in macrophages alters the expressions of inflammatory cytokines under secondary stimulation. (A)** Schematic of *ex vivo* trained immunity experimental setup (n = 3). After treatment, peritoneal macrophages were obtained and rest overnight before secondary stimulation. **(B)** Fold changes of inflammatory cytokines were determined by quantitative PCR at 6 h after macrophages treated with *E. coli^a^*. **(C)** Cytokines production in cells was determined by ELISA at 24 h after LPS treatment. **(D)** Schematic of *in vitro* trained immunity experimental setup. **(E)** Fold changes of inflammatory cytokines were determined by quantitative PCR at 6 h after LPS stimulation. Data are shown as mean ± SEM (n = 3). *P < 0.05; **P < 0.01; ***P < 0.001.

Trained immunity results in strong reaction of the innate immune system under sterile triggers of inflammation or secondary infections, enhancing protection against tumors 37-38 and sepsis 39-40. To evaluate whether BSNPs could induce trained immunity in macrophages, peritoneal macrophages and bone marrow-derived macrophages (BMDM) were obtained from the trained immunity mice and then stimulated with *E. coli^a^ ex vivo* (Figure 3A, Supplementary Figure S2A). Consistent with the previous study 41-42, β-glucan treatment enhanced TNFα, IL-1β, and IL-6 mRNA expression (Figure 3B). SPIO treatment had little effect on these inflammatory factors, while BSNP treatment significantly raised their mRNA expression (Figure 3B, Supplementary Figure S2B). Moreover, TNFα, IL-1β, and IL-6 were significantly higher inside BSNP-treated cells than other treatments (Figure 3C, Supplementary Figure S2C), suggesting that BSNP treatment enhanced the production of pro-inflammatory cytokines by macrophages. Similar results were obtained using the macrophage cell line Raw264.7 (Figure 3D-E). Collectively, these data indicated that BSNP-induced trained immunity could induce the expression of inflammatory cytokines by macrophages, which could be a powerful defense strategy against bacterial infection.

### BSNP-induced trained immunity enhances phagocytosis by macrophages

**Figure 4:**
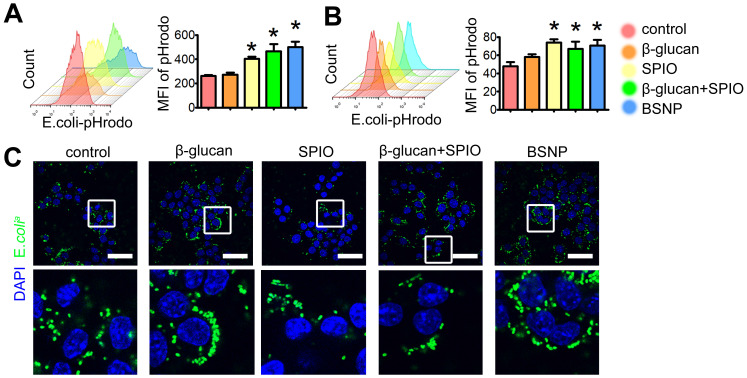
** Trained immunity promotes macrophages to phagocytize bacteria.** Mice and Raw264.7 cells were treated as described in Materials and methods section. After treatment, macrophages were obtained and rest overnight before co-cultured with pHrodo labeled *E. coli^a^* for 1 h. Phagocytosis of **(A)** peritoneal macrophages from each group was determined by flow cytometry. Phagocytosis of Raw264.7 cells from each group was determined by **(B)** flow cytometry and **(C)** confocal microscope. Scale bars, 30 µm. Data are shown as mean ± SEM (n = 3). *P < 0.05.

Besides inflammatory cytokines, phagocytosis by macrophages is another defense strategy against bacterial infection, while impaired macrophage phagocytosis is often related to poor prognosis of sepsis 43,44. Therefore, we tested phagocytosis by peritoneal macrophage from each group to further explore the superiority of BSNPs. *E. coli^a^* were stained with pHrodo, and co-cultured with macrophages for 1 h. Fluorescence green appeared only when bacteria were phagocytosed by macrophages. Mice and Raw264.7 cells were treated the same as described before (Figure 3A, 3D). Results showed that BSNP treatment promoted macrophages to phagocytose* E. coli^a^*. The mean fluorescence intensity of BSNP and the combined treatment groups was much stronger than the β-glucan or SPIO group (Figure 4A). Similar results were also obtained from the trained immunity model *in vitro* (Figure 4B). Moreover, immunofluorescence images showed more *E. coli^a^* in BSNP-treated macrophages (Figure 4C).

These data indicated that BSNP treatment could induce macrophages to engulf more pathogens. Therefore, we co-cultured BMDMs from mice with E. *coli*-pHrodo for 1 h to test primary phagocytosis. Subsequently, the cells were washed 3 times and cultured with fresh medium for another 2 h to monitor the digestion of bacteria. Fluorescence intensity was higher in the BSNP group than the control group at 1 h, indicating that BSNP promoted macrophages to phagocytose bacteria. Fluorescence intensity in the BSNP group was lower after another 2 h because BSNPs enhanced the digestion of macrophages (Supplementary Figure S2). Collectively, our results indicated that BSNPs could train macrophages into a more active state.

### BSNP-induced trained immunity prevents mice against sepsis

**Figure 5:**
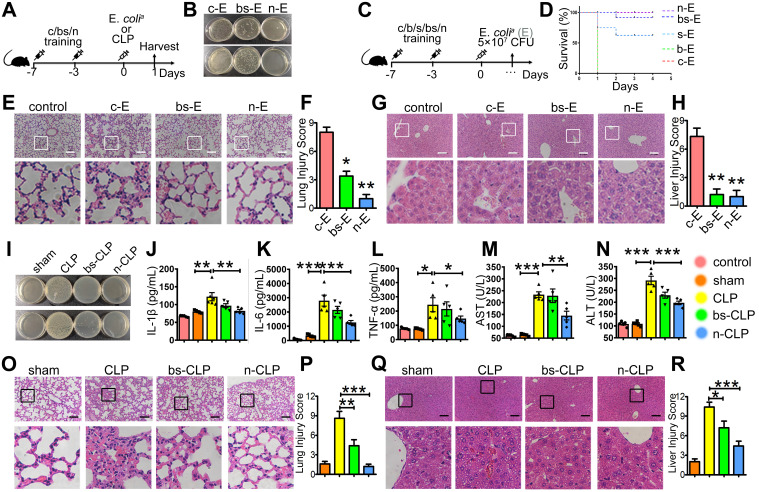
** BSNP-induced trained immunity prevents mice against sepsis. (A)** Schematic of *in vivo* trained immunity experimental setup (n = 5). **(B, I)** Blood (upper) and peritoneal lavage fluid (below) from mice 24 h after infection was plated for 16 h. Representative plate shows bacterial colonies. **(C)** Schematic for survival curve experimental setup (n = 9). **(D)** Survival curve of mice from each group after injected with lethal dose of *E. coli^a^*. Representative H&E staining of **(E, O)** lung sections or **(G, Q)** liver sections. Scale bars, 100 µm. Histological injury of **(F, P)** lungs and **(H, R)** livers in different groups scored as described in Materials and Methods. **(J-L)** Cytokines levels in serum was determined by ELISA. **(M, N)** Biochemical analysis of serum concentrations of ALT and AST. Data are shown as mean ± SEM (n = 5) *P < 0.05; **P < 0.01; ***P < 0.001.

Next, we aimed to investigate the preventive effect of BSNPs. Mice were treated with BSNPs (group n-E) or β-glucan combined with SPIO (group bs-E), followed by the intraperitoneal injection with* E. coli^a^*. We used a higher dose of *E. coli^a^* to better judge the therapeutic difference between BSNP treatment and combined treatment (Figure 5A). The BSNP-treated mice had a less bacterial burden in the blood and peritoneum than any other group (Figure 5B). BSNP treatment decreased incrassation and hyperemia in the alveolar walls (Figure 5E-F) and decreased sinusoidal congestion in the liver (Figure 5G-H). Importantly, when injected with a lethal dose of *E. coli^a^*, BSNPs treatment improved the survival rate to 100% (Figure 5C-D). These data indicated that BSNPs were superior to β-glucan and SPIO in preventing mice against *E. coli*-induced sepsis.

To further confirm the superior effect of BSNPs, we used the well-established cecal ligation and puncture (CLP) sepsis model. Mice treated with BSNPs (N-CLP) or β-glucan combined with SPIO (BS-CLP) were sacrificed at 24 h after CLP operation. Mice maintained a stable body weight during modeling while losing weight after CLP (Supplementary Figure S3). Also, mice treated with BSNPs had a less bacterial burden in blood and peritoneum than the CLP and BS-CLP groups (Figure 5I), and the TNFα, IL-1β, and IL-6 levels were elevated in the sera of CLP mice. While IL-1β and IL-6 were slightly decreased in the BS-CLP group, there was a remarkable decrease in the BSNP group (Figure 5J-K), and the TNFα level also showed the same tendency (Figure 5L). BSNP treatment decreased incrassation, hyperemia, and exudates in the alveolar walls (Figure 5O-P) and decreased sinusoidal congestion, vacuolization, and infiltration of neutrophils in the liver (Figure 5Q-R). The biochemical analysis also showed that liver damage markers, such as aspartate aminotransferase (AST) and alanine aminotransferase (ALT), were significantly decreased in the BSNP group (Figure 5M-N). These data confirmed that BSNP-induced trained immunity could better prevent mice from sepsis.

### BSNP-induced trained immunity protects mice against secondary infection

**Figure 6:**
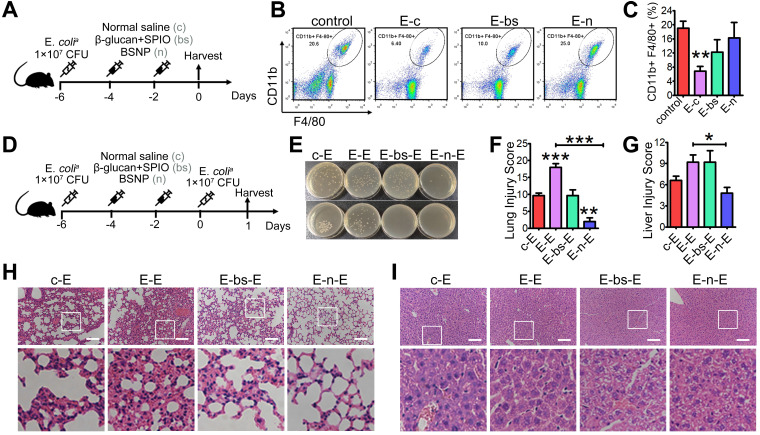
** BSNP-induced trained immunity protects mice against secondary infection. (A)** Schematic of *in vivo* phagocytic test experimental setup (n = 3). **(B-C)** Macrophages (CD11b+, F4/80+ subset) in peritoneal cells from each group were determined by flow cytometry. **(D)** Schematic of double-infection experimental setup (n = 5). **(E)** Blood (upper) and peritoneal lavage fluid (below) from mice 24 h after secondary infection was plated for 16 h. Representative plate shows bacterial colonies. Representative H&E staining of** (H)** lung sections or **(I)** liver sections. Scale bars, 100 µm. Histological injury of **(F)** lungs and **(G)** livers in different groups scored as described in Materials and Methods. Data are shown as mean ± SEM (n = 5). *P < 0.05; **P < 0.01; ***P < 0.001.

Inflammation in sepsis results in ablation of resident macrophages such as peritoneal macrophages 45-46, and depletion of macrophages is associated with poor prognosis 47-48. Also, patients with sepsis are more sensitive to secondary infections, resulting in high mortality and recurrence rate 49-50. To explore the therapeutic potential of BSNP on secondary infections, we established a sepsis model before treatment (Figure 6A). Peritoneal macrophages in mice were reduced at day 7 after primary infection. Macrophages were increased when mice were treated with BSNPs compared with those treated with combined β-glucan and SPIO (Figure 6B-C). Large peritoneal macrophages (LPMs) and small peritoneal macrophages (SPMs) are two main subsets of peritoneal macrophages. LPMs, originating from embryogenic precursors stay in enterocoelia under steady conditions. Conversely, SPMs, originating from peripheral monocytes, only increase under inflammatory conditions. When mice were injected with E. *coli^a^*, LPMs vanished within 2 days following which SPMs infiltrated in enterocoelia as replenishment. BSNPs promoted the transformation of SPMs to LPMs, accelerating the homeostasis of peritoneal macrophages (Supplementary Figure S4).

We established a double-infection model to better explore the BSNP effect under a clinical scenario (Figure 6D). Compared with the single-infection septic model, lung injury in the double-infection model was much more severe with thicker alveolar walls, more pulmonary hemorrhage, and added pulmonary debris. BSNP treatment remarkably promoted the clearance of bacteria (Figure 6E). The lung injury score of BSNP-treated mice was less than the double-infected mice, combined treatment mice, and single-infected mice (Figure 6F, H). Liver injury in the double-infection model was also much severe when compared with the single-infection model in terms of aggravated sinusoidal congestion and vacuolization. While the liver injury score was unchanged by the combined treatment of β-glucan and SPIO, it was remarkably reduced by BSNP treatment (Figure 6G, I). Thus, BSNPs were better able to promote macrophage recovery after primary bacterial infection and protect mice against secondary infection. Our data indicated that BSNPs were beneficial for the clearance of bacteria and reversion of immune paralysis.

### BSNP-induced trained immunity occurs in an mTOR-dependent manner

**Figure 7:**
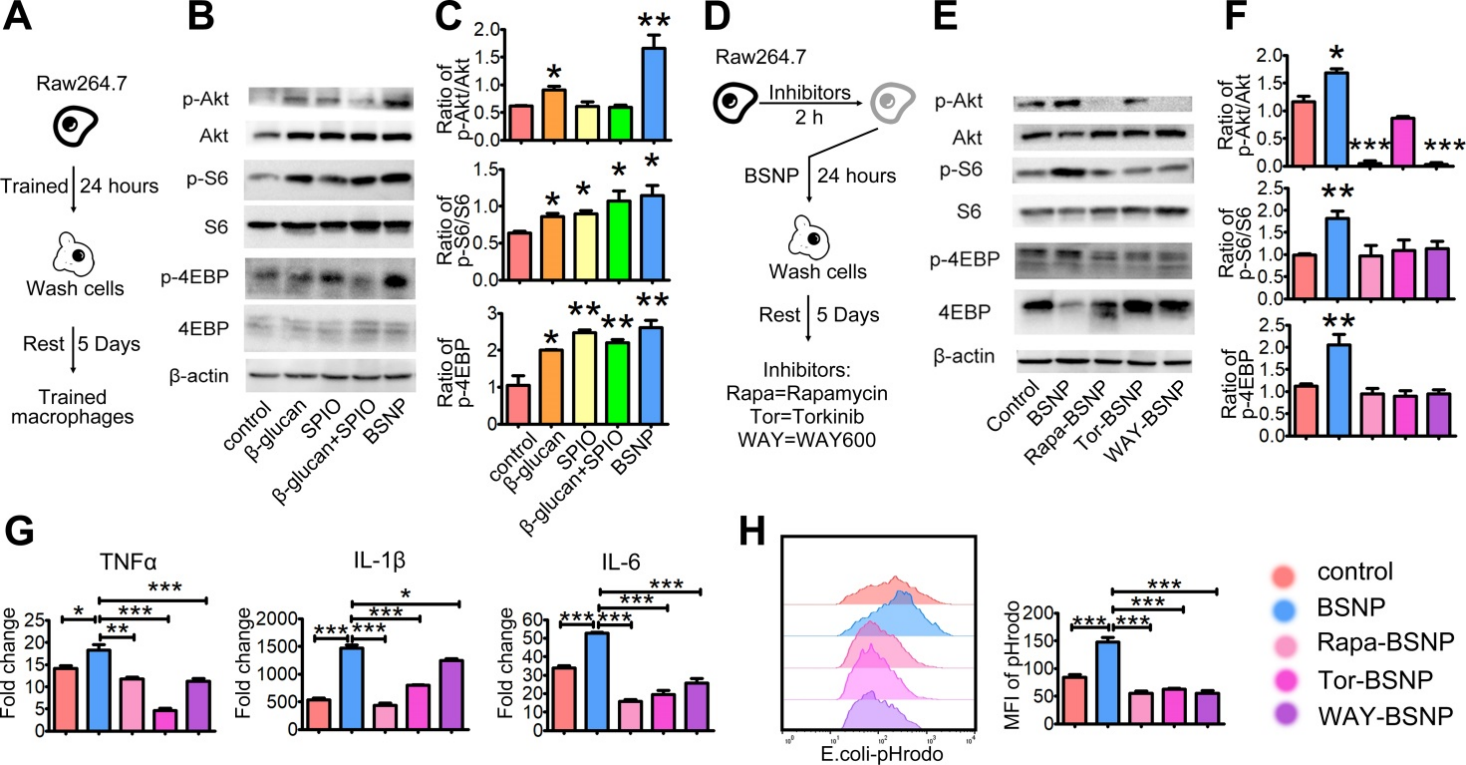
** BSNP-induced trained immunity is in an mTOR dependent manner. (A)** Schematic of *in vitro* trained immunity experimental setup. Cells were lysed after 5 days of rest. **(B-C, E-F)** Expression of proteins downstream mTOR pathway were determined by western blot. **(D)** Schematic of *in vitro* BSNP-induced trained immunity experimental setup. Raw264.7 cells were treated with mTOR inhibitor Rapamycin (10 µmol/L), Torkinib (200 nmol/L), or WAY600 (200 nmol/L) 2 h previous to BSNPs treatment. **(G)** Fold changes of inflammatory cytokines were determined by quantitative PCR at 6 h after LPS stimulation. **(H)** Phagocytosis of Raw264.7 cells from each group was determined by flow cytometry. Data are shown as mean ± SEM (n = 3). *P < 0.05; **P < 0.01; ***P < 0.001.

Previous studies have shown that the mTOR signaling pathway is central to the development of trained immunity 18. To investigate the mechanism underlying the superior therapeutic efficacy of BSNPs, we examined the phosphorylation of upstream and downstream proteins S6 and 4EBP in the mTOR pathway using the *in vitro* trained immunity model with macrophage cell line Raw264.7 (Figure 7A). Akt, S6, and 4EBP phosphorylation levels increased after BSNP treatment than after β-glucan treatment, but there was no significant difference in S6 and 4EBP phosphorylation levels between the two groups. While SPIO treatment and combined treatment with β-glucan and SPIO did not affect the phosphorylation of Akt, the phosphorylation of S6 and 4EBP was up-regulated (Figure 7B-C).

Next, we treated Raw264.7 cells with mTOR inhibitors 2 h before BSNP treatment. Rapamycin, Torkinib, and WAY600 were used to inhibit the activity of mTOR complex1, complex 1 and 2, and the assembly of mTOR complex ½, respectively (Figure 7D). BSNP-induced phosphorylation of Akt, S6, and 4EBP was inhibited by Rapamycin, Torkinib, and WAY600 (Figure 7E-F). Subsequently, Raw264.7 cells treated with mTOR inhibitors followed by BSNPs were stimulated by LPS for 6 h. The upregulation of TNFα, IL-1β, and IL-6 was significantly decreased by these inhibitors (Figure 7G). Furthermore, macrophages from each group were co-cultured with *E. coli^a^*-pHrodo for 1 h. Flow cytometry showed that BSNP-induced phagocytosis was also significantly decreased by these inhibitors (Figure 7H). These data suggested that BSNP-induced trained immunity was mTOR dependent.

**Figure 8:**
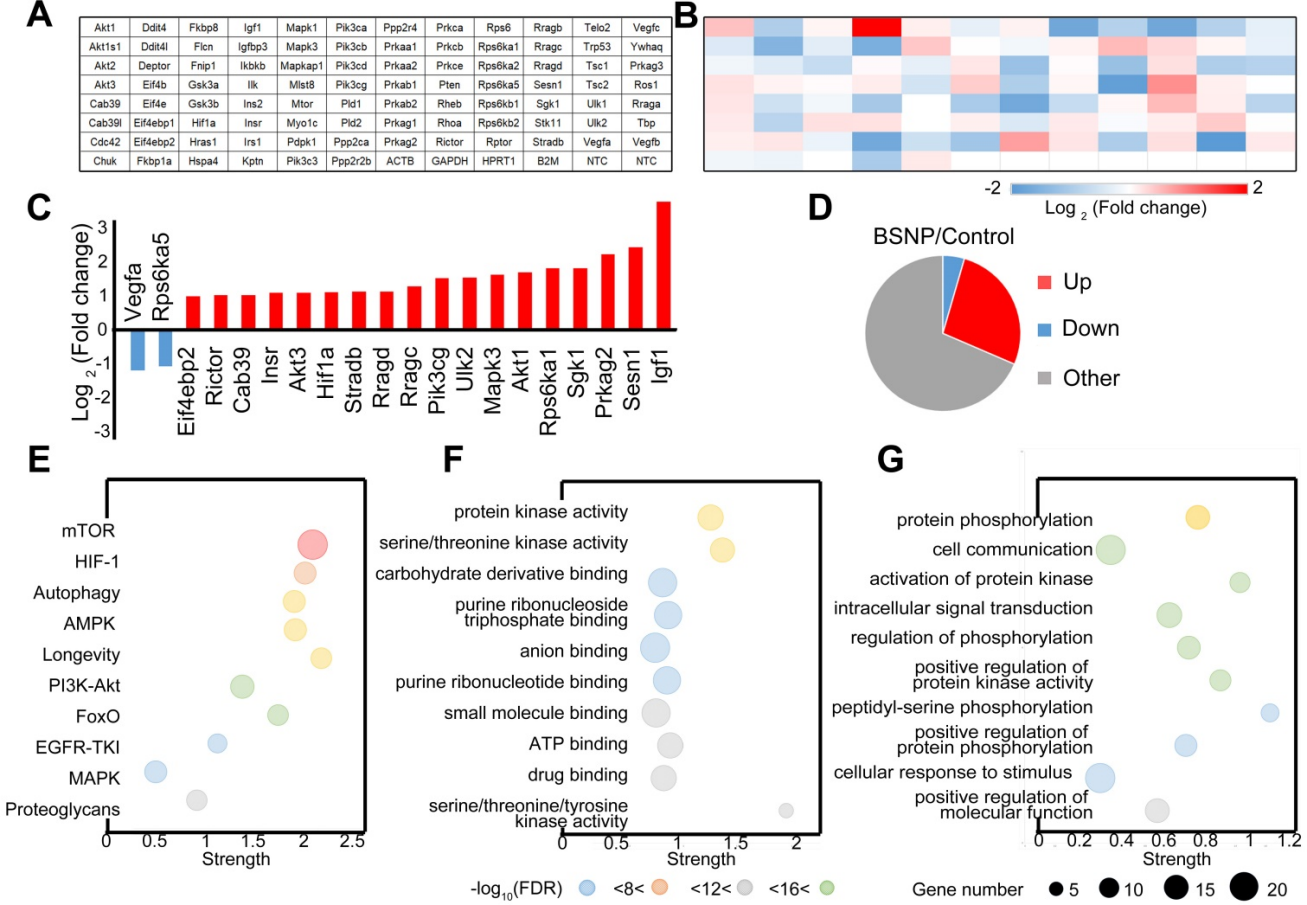
** Expression pattern of mTOR pathway related genes in trained immunity macrophages from BSNP-treated mice. (A)** Plate layout of PCR array. **(B)** Heat map showed fold changes of genes in macrophages from BSNPs treated mice versus control mice. **(C)** Major differentially regulated mTOR pathway genes. **(D)** Pie chart displayed number of differentially regulated genes. **(E-G)** Enriched GO categories of major differentially regulated genes in (E) neighborhood signaling pathway, (F) biological process and (G) functions.

Finally, we performed a PCR array on peritoneal macrophages from control mice or BSNP-treated mice to analyze the gene expression pattern in the mTOR signaling pathway (Figure 8A-B). More genes were up-regulated than down-regulated after BSNP treatment (Figure 8C). Among genes with significant fold-change (Figure 8D, Supplementary Figure S5), *Igf1* and *Sesn1* were the most up-regulated and *Vegfa* and *Rps6ka5* were the most down-regulated genes. After BSNPs treatment, active signaling pathways were analyzed using the KEGG pathway database (Figure 8E), and biological process and molecular functions were analyzed by Gene Ontology enrichment analysis (Figure 8F-G, Supplementary Figure S6).

Out data revealed a different gene expression profile in macrophages after BSNP treatment. When genes participating in mTOR signaling were investigated, *Igf1* and *Eif4ebp1* were identified as master regulators. Besides the mTOR signaling pathway, BSNP treatment also activated AMPK, PI3K/Akt, and proteoglycan signaling pathways. Thus, BSNP treatment mainly regulated cellular signal transduction, protein modification, and cell cycle progression via regulating ATP binding and the kinase activity.

## Discussion

As an emerging drug delivery system, nanoparticles have unlimited potential. It has been shown that SPIO engineered to escape the immune recognition could be less taken up by macrophages, thereby lengthening the retention time of SPIO 51 and reducing the inflammation induced by SPIO 52. Combining small molecule drugs or antibodies with SPIO could reduce their toxicity and increase their efficacy 53. In the present study, we successfully synthesized novel BSNPs by coupling β-glucan and SPIO, significantly reducing the dose of β-glucan from 500 μg per mouse to 22.5 μg on BSNPs per mouse for inducing trained immunity and lowering the possible toxicity.

We performed *in vitro* and *in vivo* experiments and found that BSNP treatment was superior to β-glucan in promoting macrophages to produce cytokines *in vivo* but not *in vitro*. Macrophages from BSNP-treated mice produced more inflammatory cytokines than β-glucan-treated mice, but there was no significant difference in Raw264.7 cells between BSNP and β-glucan. These observations indicated that BSNP uptake by peritoneal macrophages in mice was improved compared to cultured macrophages due to the coupling of β-glucan with SPIO and also because the characteristics of peritoneal macrophages were different from Raw264.7 cells. Thus, our study showed that nanoparticles were easier for uptake by macrophages *in vivo* than *in vitro*.

While exploring the mechanism of BSNP-induced trained immunity, we examined the hepcidin-ferroportin iron metabolism pathway closely associated with inflammation, infection 54-55 and sepsis 56-57. Although the β-glucan treatment had little effect on ferroportin1, SPIO could significantly raise ferroportin1 (FPN1) expression (Supplementary Figure S7A). However, in our study, treatment with β-glucan, SPIO, or combined β-glucan and SPIO protected FPN1-deficient mice against sepsis, manifested by reduced blood bacterial burden and lung damage (Supplementary Figure S7B-E). These results suggested that β-glucan and SPIO treatment effects were independent of the classical iron metabolism pathway. Also, lipocalin-2 is considered as a standby iron transporter under FPN1 deficiency 58. In lipocalin-2-deficient mice, brain injury caused by LPS was severer than normal mice 59. However, lipocalin-2 protein expression and secretion were not different among β-glucan-, SPIO-, combined β-glucan-SPIO-, or BSNP-treated macrophages (Supplementary Figure S8). These data suggested that BSNP-induced trained immunity was likely independent of lipocalin-2. Thus, the link between iron metabolism and trained immunity remains unclear.

Previous studies reported that β-glucan-induced trained immunity induced macrophages to produce more inflammatory cytokines under secondary stimulation 60-61. Inflammation is a double-edged sword, as it results in tissue injury but is also necessary for host defense. We speculated that BSNPs could serve as an immunostimulatory agent, protecting the host against primary and secondary infections 62-63. Activation and M1 polarization of macrophages are beneficial for bacterial clearance and survival rate of infected mice 64-65. The way to balance inflammation and antibacterial activity of macrophages needs to be further studied.

In the present study, we found that inflammatory cytokine levels inside and outside the macrophages were different. After LPS stimulation for 24 h, inflammatory cytokine levels in the supernatant of macrophages obtained from BSNP-treated mice were similar to non-treated mice (Supplementary Figure S9). The data (Figure 3) suggested that BSNP-induced trained immunity could induce macrophages to produce but not secrete inflammatory cytokines. Furthermore, after treatment for 6 days, β-glucan induced M1 polarization, while combined β-glucan and SPIO and BSNPs reduced M1 polarization. On the contrary, β-glucan, SPIO, combined β-glucan and SPIO, or BSNPs had little effect on M2 polarization (Supplementary Figure S10). It is conceivable that the BSNP effect on macrophage polarization is conducive to killing bacteria inside macrophages while reducing the damage to adjacent cells. In summary, our study provided strong evidence for increased macrophage phagocytosis of bacteria via BSNP-induced trained immunity.

## Conclusions

We successfully synthesized novel nanoparticles, BSNPs, by coupling β-glucan and SPIO and found that BSNPs could be taken up by macrophages and induced trained immunity efficiently. BSNPs induced macrophages to produce inflammatory cytokines and accelerated the clearance of bacteria by phagocytosis. BSNP-induced trained immunity could guard against sepsis and also protect against re-infection in the short term. The BSNP-induced trained immunity functioned in an mTOR-dependent manner. Our study provided a new prevention and treatment strategy for sepsis. Furthermore, it offered a theoretical basis for the application of nanomaterials in trained immunity that could be extended to other situations such as severe trauma, pneumonia, and systemic lupus erythematous.

## Supplementary Material

Supplementary figures and tables.

## Abbreviations

SPIO: superparamagnetic iron oxide
BSNPs: β-glucan coupling SPIO nanoparticles
mTOR: mammalian target of rapamycin
qRT-PCR: quantitative real-time polymerase chain reaction
COVID-19: coronavirus disease 2019
FDA: Food and Drug Administration
IL: interleukin
TNF: tumour necrosis factor
LPS: lipopolysaccharide
RhB: Rhodamine B isothiocyanate
EDC: 1-ethyl-3-(3-dimethylaminopropyl) carbodiimide hydrochloride
DMEM: Dulbecco's modified Eagle's medium
ELISA: Enzyme Linked Immunosorbent Assay
SDS-PAGE: sodium dodecyl sulfate polyacrylamide gel electrophoresis
PVDF: poly(1,1-difluoroethylene)
PFA: paraformaldehyde
H&E: hematoxylin-eosin
EtOH: ethyl alcohol
MFI: mean fluorescence intensity
DAPI: 4,6-diamino-2-phenyl indole
CD11b: integrin Alpha M
F4/80: mouse EGF-like module-containing mucin-like hormone receptor-like 1
BMDM: bone marrow derived macrophages
LPM: Large peritoneal macrophages
SPM: small peritoneal macrophages
