# Supplementary material for: β-glucan-coupled superparamagnetic iron oxide nanoparticles induce trained immunity to protect mice against sepsis
Source: Theranostics. 2022 Jan 1;12(2):675–88. doi: 10.7150/thno.64874 (PMC8692910; doi:10.7150/thno.64874)
Supplement: Supplementary file 1 — Supplementary figures and tables. [file thnov12p0675s1.pdf]

Supplementary material

Table S1. Antibody used for flow cytometry

Name	Brand	Cat. No.
APC anti-mouse F4/80 Antibody	BioLegend	123115
PE/Cyanine7 anti-mouse/human CD11b Antibody	BioLegend	101216
APC Rat IgG2a kappa Isotype Control	Invitrogen	17-4321-81
PE/Cyanine7 Rat IgG2a kappa Isotype Control	Invitrogen	25-4321-82

Table S2. Standard curve used for quantitative analysis

Concentration	Peak Area
0.025	2714682
0.05	5361935
0.1	10979598
0.2	21780923
0.4	39852190

Table S3. Antibody used for western blot

Protein	Brand	Cat. No.
Akt	Cell Signaling Technology	4691S
p-Akt	Cell Signaling Technology	4060P
S6	Cell Signaling Technology	2217S
p-S6	Cell Signaling Technology	4858S
4EBP	Cell Signaling Technology	9644S
p-4EBP	Cell Signaling Technology	2855S

Table S4. Sequences of forward and reverse primers used for PCR amplification

Gene	Forward Primer	Reverse Primer
β-actin	AGGTGACAGCATTGCTTCTG	GGGAGACCAAAGCCTTCATA
IL-1β	TTCAGGCAGGCAGTATCACTC	GAAGGTCCACGGGAAAGACAC
TNFα	CCCCAAAGGGATGAGAAGTT	CACTTGGTGGTTTGCTACGA
IL-6	CTGCAAGAGACTTCCATCCAG	AGTGGTATAGACAGGTCTGTTGG

Table S5. Evaluation of particle sizes, PDI and zeta potential for nanoparticles

Sample	Particle Sizes (nm)	PDI	Zeta Potential
SPIO	27.48 ± 0.13	0.234	-41.69 ± 1.36
BSNP	29.46 ± 0.24	0.253	-34.37 ± 1.45

Supplementary Figure

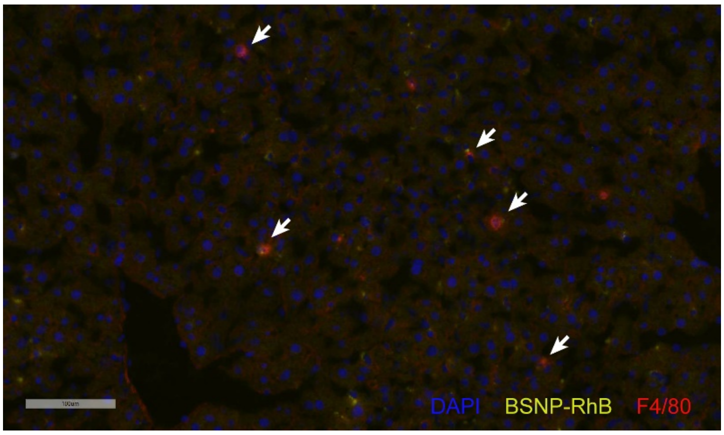

Supplementary Figure S1. Immunofluorescence of macrophages and BSNP-RhB in liver. Mice were injected intraperitoneally with BSNP-RhB and sacrificed at 24 h. Scale bar, 100 μm.

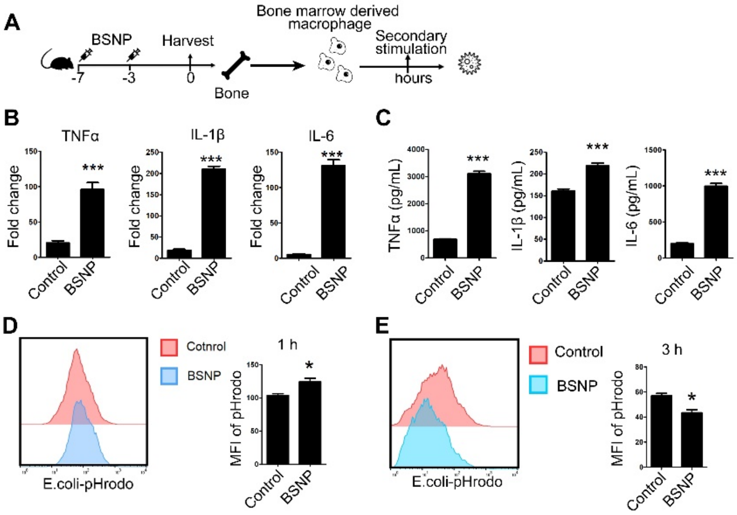

Supplementary Figure S2. BSNP-induced trained immunity enhances immune responses in macrophage. (A) Schematic of ex vivo trained immunity experimental setup (n = 3). Bone marrow was obtained and derived into macrophages with M-CSF treatment. (B) Fold changes of inflammatory cytokines were determined by quantitative PCR at 6 h after macrophages treated with LPS. (C) Cytokines production in the cells was determined by ELISA at 24 h. (D) Bone marrow derived macrophages were co-cultured with pHrodo labeled *E. coli*[a] for 1 h, (E) then washed 3 times, replaced with fresh culture medium, and then rest for another 2 h. Phagocytosis

of Raw264.7 cells from each group was determined by flow cytometry. Data are shown as mean ± SEM (n = 3). *P < 0.05; ***P < 0.001.

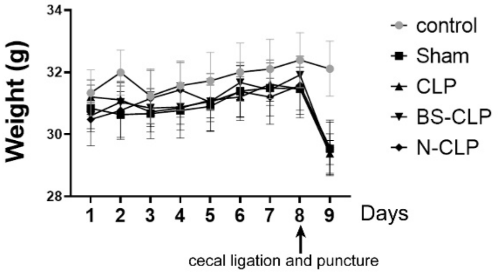

Supplementary Figure S3. Mice weight during experiment. Mice were treated with combined β-glucan or BSNPs before CLP modeling. Mice weight (g) was recorded every day until sacrifice. Data are shown as mean ± SEM (n = 5).

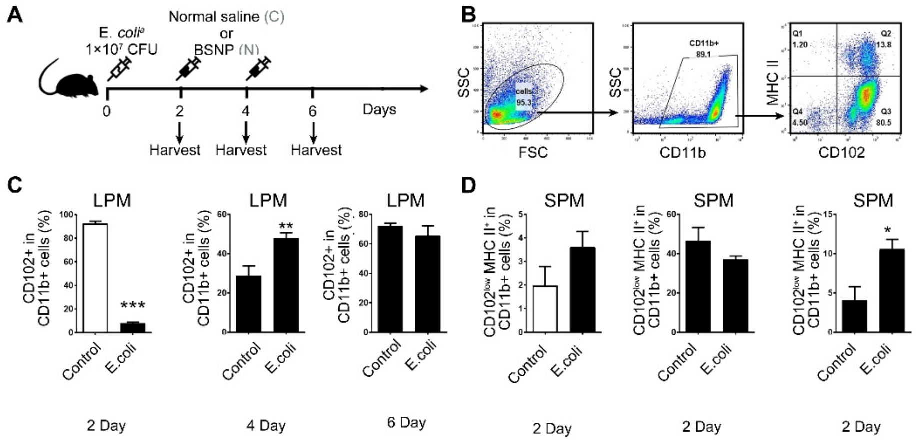

Supplementary Figure S4. Subsets of peritoneal macrophages from mice. (A) Schematic of in vivo phagocytic test experimental setup. (B) Identification of large peritoneal macrophages (LPM) and small peritoneal macrophages (SPM) based on CD102 and MHC II. Flow cytometry showed the percentage of (C) LPM and (D) SPM at different time after mice injected with E. *coli*[a]. Data are shown as mean ± SEM (n = 3). *P < 0.05, **P < 0.01; ***P < 0.001.

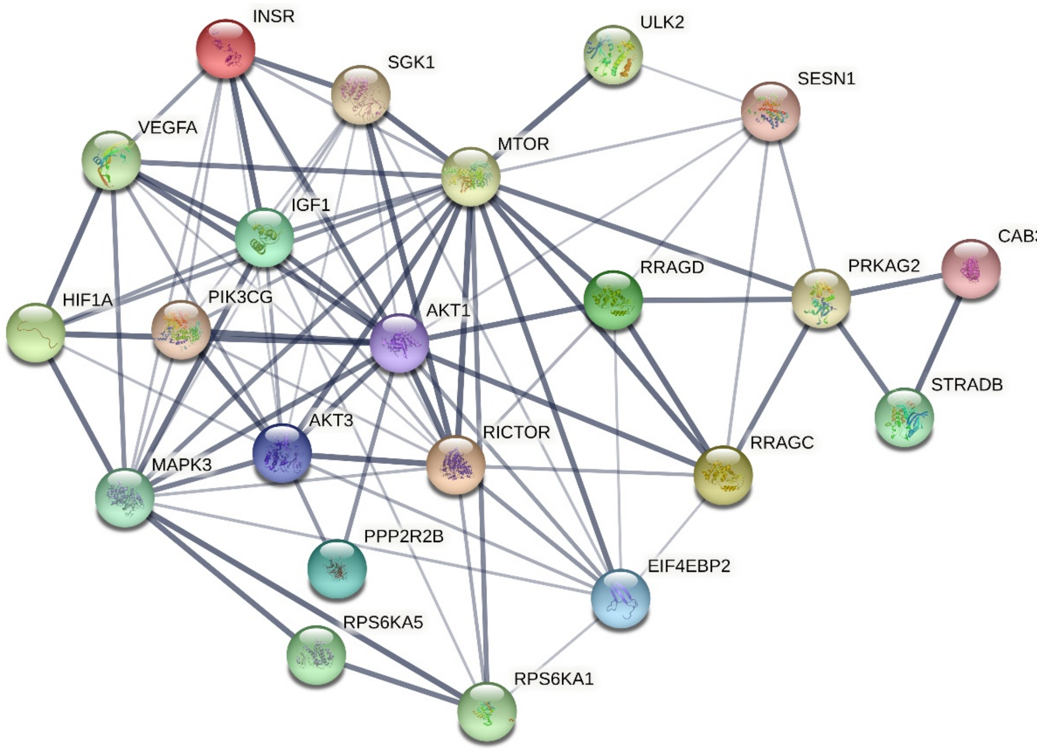

Supplementary Figure S5. STRING interaction pathway of major differentially regulated mTOR pathway genes.

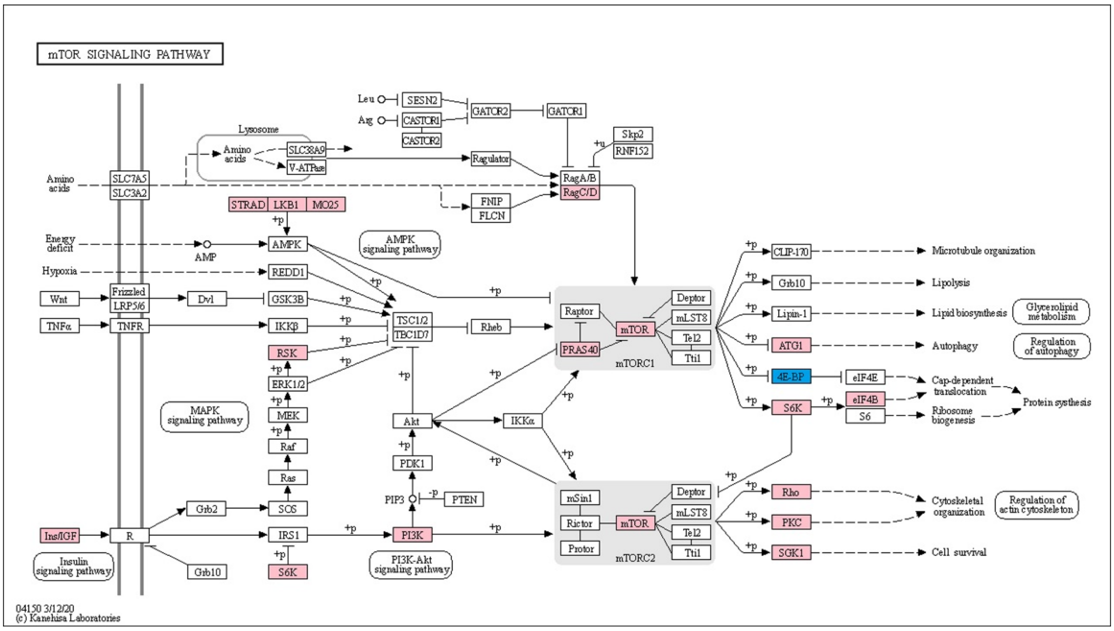

Supplementary Figure S6. Schematic representation of up-regulated genes (red) and down-regulated genes (blue) in mTOR signaling pathway of macrophages from BSNP-treated mice.

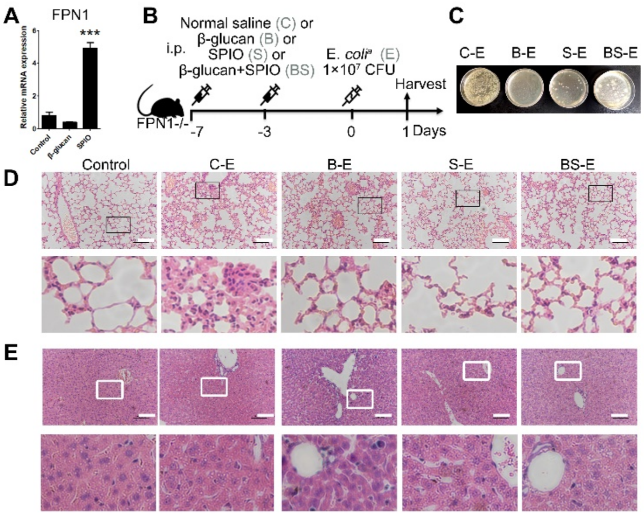

Supplementary Figure S7. Trained immunity protects FPN1-/- mice against sepsis. (A) Raw264.7 cells were treated with β-glucan or SPIO for 24 h. Relative mRNA expression of FPN1 was determined by quantitative PCR. (B) Schematic of sepsis after trained immunity experimental setup (n = 5). (C) Blood from mice 24 h after infection was plated for 16 h. Representative plate shows bacterial colonies. (D-E) Representative H&E staining of (D) lung sections or (E) liver sections. Scale bars, 100 μm. ***P < 0.001.

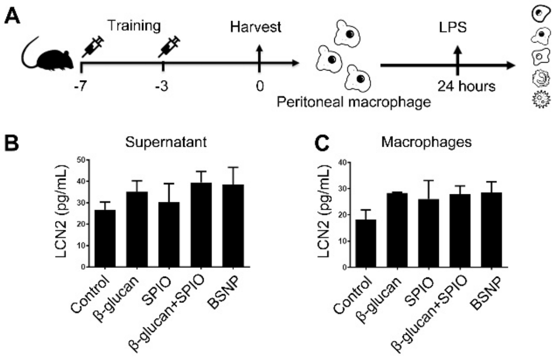

Supplementary Figure S8. Lipocalin2 (LCN2) expression in macrophages after trained immunity. (A) Mice were treated with β-glucan, SPIO, combined β-glucan and SPIO or BSNPs as described in Materials and methods. Expression of LCN2 in supernatant (B) and lysate (C) of peritoneal macrophages from trained mice was determined by ELISA. Data are shown as mean ± SEM (n = 3).

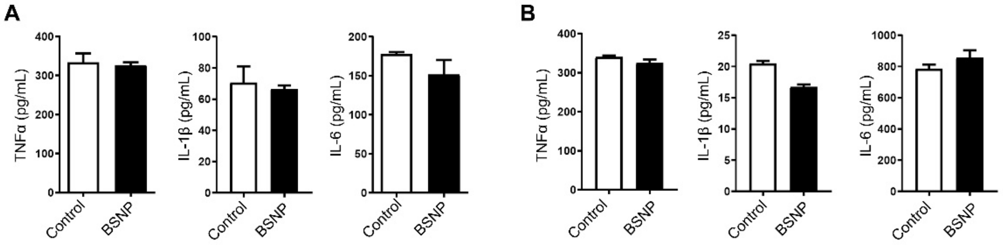

Supplementary Figure S9. Cytokine secretion of BSNP-trained macrophages. Mice and Raw264.7 cells were treated with BSNPs in the same way as in Figure 3. (A) Peritoneal macrophages from BSNP-treated mice and (B) Raw264.7 were stimulated with LPS for 24 h. Production of TNF-α, IL-1β and IL-6 in supernatant were determined by ELISA. Data are shown as mean ± SEM (n = 3).

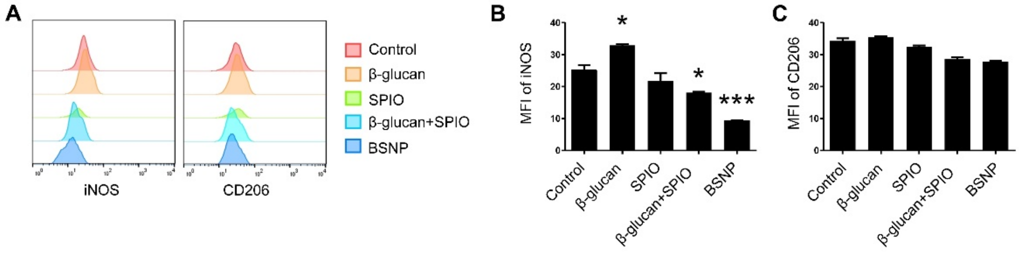

Supplementary Figure S10. Polarization of trained macrophages. Raw264.7 cells were treated with β-glucan, SPIO, combined β-glucan and SPIO or BSNPs as described in Materials and methods. Polarization of trained Raw264.7 cells were measured by flow cytometry. Data are shown as mean ± SEM (n = 3). *P < 0.05; ***P < 0.001.
